# Analyzing the Relationship Between Bus Pollution Policies and Morbidity Using a Quasi-Experiment

**DOI:** 10.1097/MD.0000000000001499

**Published:** 2015-09-18

**Authors:** Nicole S. Ngo

**Affiliations:** From the Department of Planning, Public Policy, and Management, 1209 University of Oregon, Eugene, OR 97403-1209.

## Abstract

Transit buses are used by millions of commuters every day, but they emit toxic diesel fumes. In 1988, the U.S. Environmental Protection Agency implemented emission standards for transit buses, which have been continually updated. Yet there is no quantitative evidence of the health benefits from these bus pollution policies due to data constraints and confounding variables.

In this study, a quasi-experiment is used to exploit the geographic and temporal variation in emission standards by using bus vintage as a proxy for bus emissions. This is accomplished using a unique, rich panel data set, which includes daily information on bus vintage and route for the New York City Transit bus fleet between 2006 and 2009. This information is merged with daily data on emergency department (ED) visits for respiratory illnesses, which include patients’ residences at the census block level and exact admission date. Economic benefits resulting from these bus pollution policies are then estimated.

Results show that stricter transit bus emission standards by the U.S. Environmental Protection Agency for particulate matter are associated with reduced ED visits for respiratory diseases for patients living within a few hundred feet of a bus route. These findings demonstrate that bus pollution policies have made critical improvements to public health.

## Abstract 2

Supplemental Digital Content is available in the text

## INTRODUCTION

Transit buses are a common feature in cities. In the United States, ∼10.4 million transit bus passenger trips were taken on an average weekday in 2011.^1^ However, buses use diesel fuel and numerous studies show exposure to diesel emissions has negative effects on human health, such as respiratory disease.^2,3^ In 1988, the U.S. Environmental Protection Agency (EPA) started regulating transit bus emissions of nitrogen oxides (NOx) and particulate matter (PM). These policies have been continually updated, whereby 2010, emission standards (or caps) had reduced by 98% (Table 1).

**TABLE 1:**
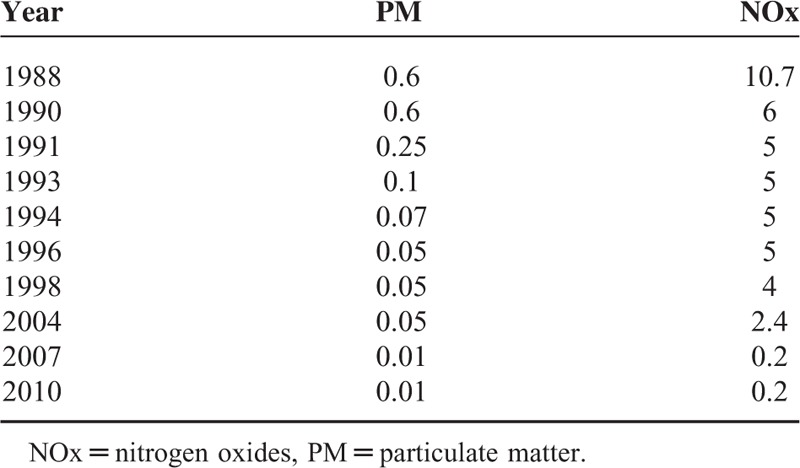
EPA Transit Bus Emission Standards (gm/bhp-h)

In New York City (NYC), bus pollution remains a critical public health issue for almost half the residents (∼4 million people) who live within a few hundred feet of a bus route. In addition to federal policies, NYC Transit (NYCT) took further steps to reduce bus emissions in 2000 when its bus fleet sought to become the cleanest in the world, including using ultra low sulfur diesel and retrofitting buses with diesel particulate filters.^4^ However, despite major progress, a 2009 study by the NYC Air Community Survey found that buses remained a major pollution source, and other studies confirmed the serious nature of this problem.^5–8^ Consequently, understanding the health impacts and cost-savings of bus pollution policies is crucial for improving public health in urban areas. In fact, a study by NYC's Department of Health and Mental Hygiene (2011) estimated that between 2005 and 2007, PM_2.5_ (PM with an aerodynamic diameter < 2.5 μm) concentrations contributed to ∼6000 emergency department (ED) visits for asthma per year.^9^ The same study suggested that a 10% reduction in PM_2.5_ could reduce annual asthma ED visits by 660 visits.

The aim of this study is to observe the relationship between bus pollution policies and morbidity, specifically respiratory disease, an illness strongly associated with air pollution. Whereas concerns over public health have motivated these bus pollution policies, no study has rigorously evaluated their health impacts due to data constraints.^10^ A common problem in observational studies examining the relationship between air pollution and health is controlling for confounding variables, which can be challenging since many factors or characteristics are unobserved and cannot be properly accounted for in regression analysis.

More recently, studies have begun to exploit natural or quasi-experiments, where treatment is assigned outside the experiment.^11–13^ In this study, a quasi-experiment is used that exploits bus vintage (ie, the year the bus was built) as a proxy for bus emissions. Bus vintage is an appealing proxy for a few reasons. First, individual exposure to bus vintage could be considered “as good as random” since the variation of bus vintage to a neighborhood or bus shift is unlikely correlated to confounding variables. Anecdotal evidence from NYCT Metropolitan Transportation Authority (MTA) employees suggest that bus drivers drive the same route, but not the same buses since bus drivers use the first bus in the bus depot prepped for that shift. Additionally, bus routes travel through several different neighborhoods, making it difficult to send older buses to only poorer neighborhoods. Other work in NYC has reinforced this, showing no relationship between the distribution of bus vintage and neighborhood characteristics, including education and race.^10^ Consequently, by associating bus vintage with bus emissions using these EPA emission standards from Table 1, the impacts of bus pollution policies on health can be statistically isolated from possibly confounding variables.

Second, the literature suggests that bus vintage is a reliable proxy for bus emissions. Previous work shows that bus vintage is one of the most important factors affecting bus emissions.^14,15^ For example, one study found that between 1995 and 2006, 89% of PM and NOx emissions reductions for the NYCT bus fleet was due to the replacement of older buses with newer buses that followed more stringent EPA emission standards.^16^

**FIGURE 1:**
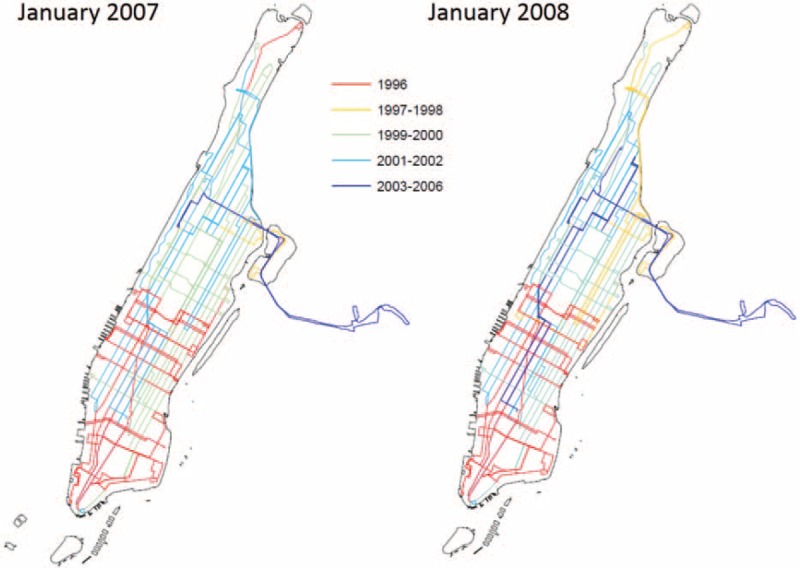
Map of geographic and temporal variation in bus vintage in Manhattan, NYC, showing the long-term variation in bus vintage.

Third, whereas detailed information on individual bus emissions is unavailable or severely limited, ample, detailed data on bus vintage is available. A unique data set of bus information was obtained through the Freedom of Information Act (FOIA) from NYCT MTA. It includes bus vintage and route at the bus shift level for a given date at major bus depots in NYC (> 20 million observations). Figure 1 is an example of the temporal and spatial variation of bus vintage in Manhattan, a borough of NYC, and Figure S2, http://links.lww.com/MD/A418 in the supplementary file is an example of the daily variation in bus vintage for route M1.

The objective of this study is to investigate the relationship between these EPA transit bus emission standards and respiratory disease in NYC using restricted data on the universe of outpatient ED visits in NYC between 2006 and 2009, which includes patients’ residences at the census block level, exact admission date, and important patient characteristics. Observing patients’ census blocks is critical as it is used to determine, which bus routes pass by patients’ residences at a high spatial resolution and minimize measurement error. Census block dummy variables are also included as controls to compare patients who reside on the same census blocks and mitigate omitted variable bias. Lastly, a back-of-the-envelope calculation estimates the economic benefits associated with savings in ED visits resulting from transit bus pollution policies for NYC residents who live near a bus route.

## METHODS

### Hospitalization Data

Restricted outpatient ED data between 2006 and 2009 from the NY State Department of Health's Statewide Planning and Research Cooperative System are used (∼3.8 million observations) with approval from the Institutional Review Board of NY State Department of Health. These data include information on principal diagnoses using the *International Classification of Diseases* (ICD-9) at NYC hospitals. The health outcomes of interest are respiratory disease (ICD9 codes 460–519) and injuries and poisonings (ICD9 codes 800–999). ICD9 codes used for acute respiratory disease were 460–478, 493–494, 500–508, 514, 516–519 and for bronchitis was 490. Data also include exact admission date and street addresses which are converted into geo-coordinates using ArcGIS and a single-line street base map dataset called “LION” from BYTES of the Big Apple.^17,18^ Health data are then aggregated from the street address to the census block level using the 2000 census GIS map of census blocks, as it covers the study period of interest, to assign patients’ exposure to nearby bus routes.^17^

### Bus Data

Bus data collected from daily maintenance NYCT bus pull-out sheets for the years 2006 to 2009 were obtained through FOIA and include bus vintage for each route and shift. Data on bus vintage, which was used as a proxy for bus emissions, were then aggregated to the census block level to determine daily average bus pollution for a given route (see section 4 of the supplementary file for more information, http://links.lww.com/MD/A418). Estimated emissions of bus PM and bus NOx are based on bus vintage using the EPA emission standards from Table 1 (eg, if a bus is built in 2007, then it is assigned bus PM emissions of 0.01 units and bus NOx emissions of 0.2 units).

### Weather Data

Data on daily maximum temperature and precipitation from the weather station in Central Park in Manhattan in NYC were collected from the National Climactic Data Center.

### Merging Bus and Health Data Sets

In 2000, there were 36,719 census blocks and 8,008,278 people in NYC, implying an average census block size of 218 people.^18^ To merge datasets, ArcGIS is used to overlap maps of 2008 bus routes with 2000 census block data to determine which routes are within 300 ft of a centroid of each census block. Only census blocks that have a centroid within 300 ft of a bus route are kept, which is 47% of all NYC blocks. These data are then merged with the bus data obtained from FOIA based on the date and the bus route. However, to reduce the measurement error, census blocks are dropped if data for all bus routes are unavailable for that date. This reduces the sample size dramatically to 13% of the original data set. Finally, estimated daily average emissions of bus PM and NOx for each census block are merged with admission date and census block from the restricted hospitalization dataset (see section 4 of the supplementary file for more information, http://links.lww.com/MD/A418).

### Statistical Analysis

This study uses a quasi-experiment and panel data to statistically isolate the impact of bus emissions on ED visits for respiratory disease. An ordinary least squares (OLS) framework is used and focuses on patients who reside within 300 ft (∼91 m.) of a bus route as previous work suggests that street-level pollution from diesel vehicles reach background levels at this distance.^19^ As bus emissions are not directly observed, the terms “bus PM,” “bus NOx,” or “bus pollution” refer to estimated bus emissions using Table 1, unless otherwise noted. Associations with bus vintage are also examined to offer insight into the importance of local policies and have implications regarding the generalizability of these results to other diesel vehicles, such as trucks. Below is the baseline regression for the static model examining the contemporaneous relationships between bus pollution and health: 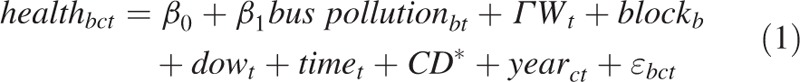


where the variable, *health*, is the number of ED admissions for patients who reside on block *b* in neighborhood or community district (CD) *c* on date *t* for a particular health outcome of interest. The explanatory variable of interest is the variable, *bus pollution*, which represents estimated daily average bus PM, bus NOx, or bus vintage. Weather (*W*), specifically maximum temperature and precipitation, is accounted for since it is correlated to both pollution and health. Dummy variables for each census block (*block*_*b*_) are included to control for fixed or time-invariant characteristics within each block, further eliminating biases from confounding variables. To account for changes over time, dummy variables for each day-of-week (*dow*_*t*_) are included to control for weekend versus weekday effects and an interacted dummy variable for each month-year (*time*_*t*_) to account for seasonality. Finally, CD-year trends are used to control for possibly unobserved, time-varying characteristics correlated to pollution and health, such as differing trends in socioeconomic conditions across neighborhoods. To address spatial correlation across census blocks, the error term, ε_bct_, is clustered at the CD level and accounts for correlations within the same CD (there are 71 CDs in NYC). Summary statistics of covariates and health outcomes are in Table 2. Analysis is carried out using STATA 13.^20^

**TABLE 2:**
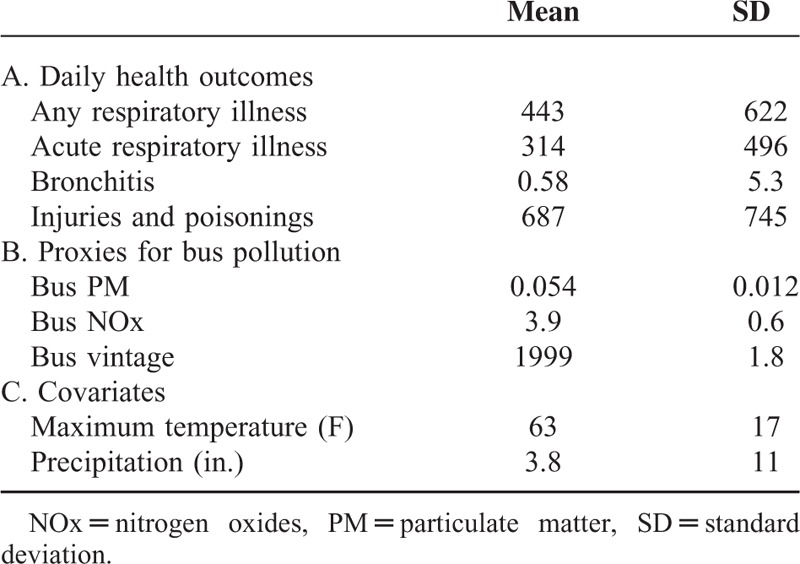
Daily Summary Statistics for the Dependent and Independent Variables (N **=** 501,779)

The coefficient of interest is β_1_, which represents the relationship between a 1 unit (gm/bhp h) increase in exposure to bus pollution, as approximated by emission standards based on bus vintage, and ED visits for the health outcomes of interest, specifically, all respiratory diseases, acute respiratory illness (which includes asthma), or bronchitis. The variation in bus pollution across days and routes, which could be considered “as good as random” due to the quasi-experiment nature of this study, is used to determine the relationship between health and bus emissions. To avoid problems of multicollinearity, associations of bus PM, bus NOx, and bus vintage are first examined separately with health.

It is expected that an increase in bus PM or bus NOx will be positively associated with the number of ED visits for respiratory illnesses. However, an increase in bus vintage should be associated with a decrease in ED visits since a marginal increase in bus vintage represents the impact of exposure to a newer, cleaner bus. Associations with injuries and poisonings are also observed as a falsification test and should be insignificant.

## RESULTS

### Health Impacts of Bus Pollution Policies

Results of Eq. (1) are in column 1, Table 3 where each coefficient is from a different regression and standard errors are in brackets below each coefficient (^∗^*P* < 0.05, ^∗∗^*P* < 0.01) (N = 501,779). These coefficients represent the marginal impact of a change in estimated bus pollution on ED visits for different categories of respiratory disease. Estimates in column 1 show that a 0.1 unit increase in bus PM (bus PM never exceeds 1 unit) corresponds to a statistically significant 0.025 increase in daily ED visits for all respiratory diseases (Panel A), 0.021 increase in acute respiratory disease (Panel B), and a 0.0049 increase for bronchitis (Panel C). Results also show a statistically significant decrease for all respiratory diseases from exposure to newer buses. As a falsification test, the impact of bus pollution on injuries and poisonings is observed (Panel D) and shows no effect, as expected.

**TABLE 3:**
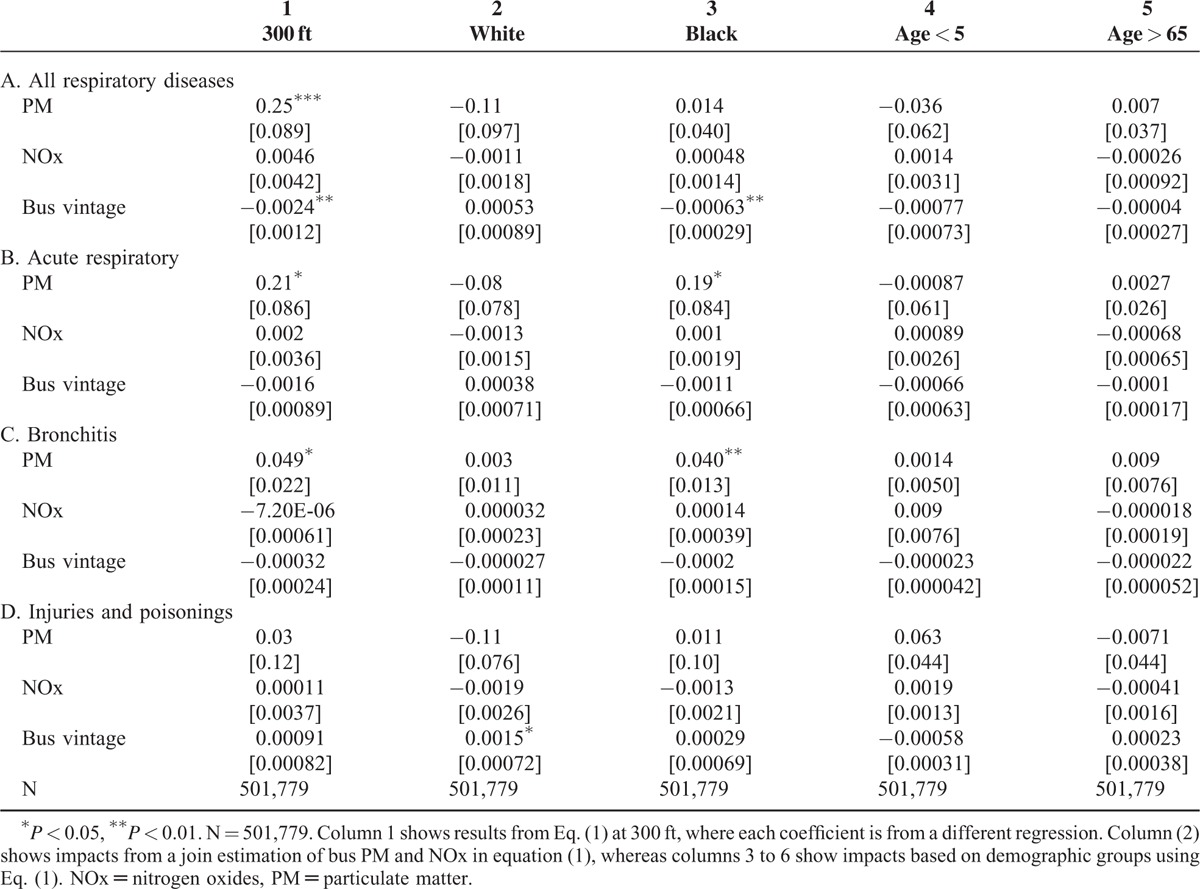
Contemporaneous Relationship Between Bus Pollution on the Number of ED Visits for Different Respiratory Illnesses at 300 ft Using the Static Model

Differences in health outcomes may also vary by the socioeconomic status or other environmental factors, though there is not enough information to directly test for this. Instead, impacts by demographic group are observed, specifically race and age using Eq. (1). First, relationships among Whites and Blacks are investigated (Table 3). There are no statistically significant associations among the health outcomes of interest for Whites (column 2) from bus pollution, but there is a significant positive relationship among Blacks (column 3) from exposure to bus PM with ED visits for acute respiratory diseases and bronchitis. Findings also show a small significant, negative relationship with exposure to bus vintage. Next, associations by age groups are examined, in particular, children at the age of 5 years and under or elderly individuals ≥65, as the literature suggests that these groups are more vulnerable to poor air quality. However, there is no statistically significant relationship with bus pollution for these age groups (columns 4 and 5, Table 3). A falsification test is also performed for these demographic groups and results show a statistically significant relationship between bus vintage and injuries and poisonings. This, however, does not change the main implications of the paper since this result is not robust across different regression models and could be due to statistical chance.

### Bus Vintage and Background Pollution Levels

**FIGURE 2:**
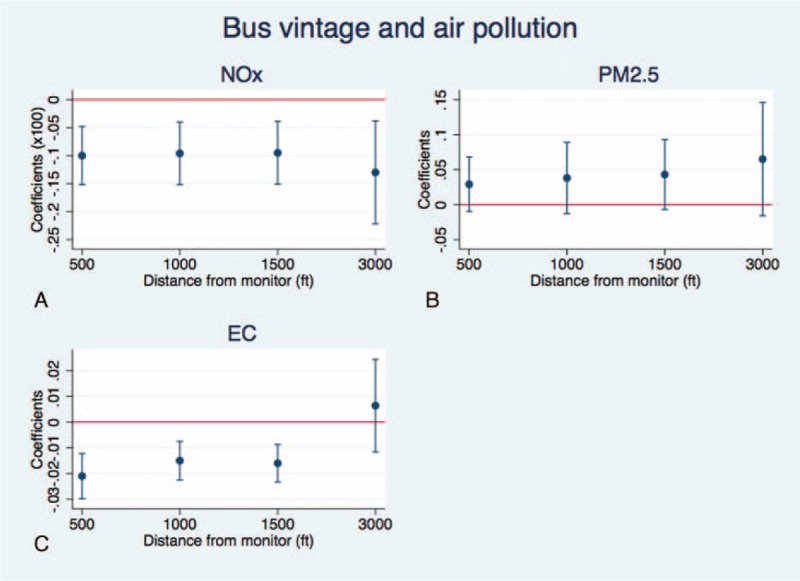
The relationship between bus vintage and background levels of air pollution in NYC for NOx, PM_2.5_, and EC at different distances, specifically 500, 1000, 1500, and 3000 ft (not to scale). Each bar represents the coefficients and its standard errors (the circle is the coefficient, and the bar around each coefficient is the standard error). The horizontal line represents 0.

Previous work shows the importance of bus vintage and emission standards on bus pollution. This is further explored in this study using the bus data obtained through FOIA as a way to assess if bus vintage is a reliable proxy for bus emissions.^10,14–16^ Of particular interest are background levels of PM_2.5_, NOx, and elemental carbon (EC), where the latter of which is a constituent of PM_2.5_ often used as proxy for diesel emissions. Bus pollution is expected to have a positive effect on PM_2.5_, NOx, and in particular, EC. A negative relationship between bus vintage and background pollution levels is expected. An OLS regression framework is used and includes controls for seasonality, long-run time trends, weather, and pollution monitor dummy variables to account for characteristics particular to a monitor (eg, height and location) (see section 2 of the supplementary file for more information, http://links.lww.com/MD/A418). As a robustness check, this relationship is explored within different distances of pollution monitors. The coefficient should decrease and become less precise with distance as the error is random and classical measurement error indicates that including routes further away will bias estimates toward zero.^21^ Results are in Figure 2, where each circle and bar represents the coefficient and its standard errors (resp.) and the horizontal line is zero. These findings show a statistically significant, negative relationship between bus vintage and background levels of NOx and EC, but not of PM_2.5_. The size of the coefficients for NOx and EC slightly decreases with distance and remains statistically significant until 3000 ft, the furthest distance.

### Sensitivity Analysis

Several sensitivity checks are performed to determine the sensitivity of the baseline results. The relationship between bus pollution and health is observed using a distributed lag model, as estimates from the static model could be biased if an ED visit today was the result of exposure to bus pollution a few days ago or that accumulated over time, where 7-day lags of bus PM, bus NOx, bus vintage, and weather are included in Eq. (1). Next, the cumulative impact over the entire 8-day period is examined since it better reflects a permanent change in bus pollution relative to only studying results from the static model in Eq. (1). The cumulative impact is estimated using the “lincom” command in STATA 13, which takes a linear combination of coefficients and adjusts standard errors appropriately.^20^ Results of the cumulative impacts are in Table 4 and show increases for all health outcomes of interest associated with increased cumulative exposure to bus PM, except for injuries and poisonings.

**TABLE 4:**
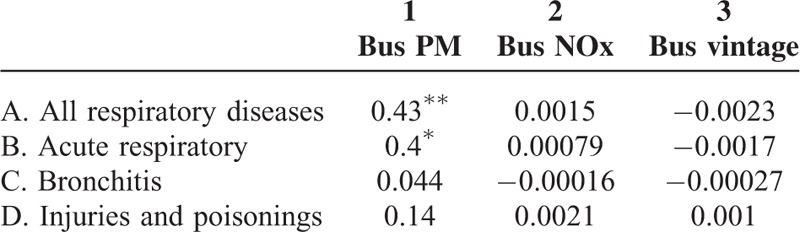
8-Day Cumulative Impact of Bus Pollution on the Number of ED Visits for Different Respiratory Illnesses at 300 ft Using the Distributed Lag Model

Another concern is that the relationships among bus PM, bus NOx, and health are observed separately, so omitted variable bias could still be a problem. Consequently, Eq. (1) is run again, but includes both bus PM and bus NOx in the same regression and results from this joint estimation are statistically similar to the baseline results in Table 3 (column 1, Table 5). As another sensitivity check, associations between bus pollution and health for bus routes within 500 ft (∼152 m) of patients’ residences are observed and results are statistically similar to the baseline results (column 2, Table 5).

**TABLE 5:**
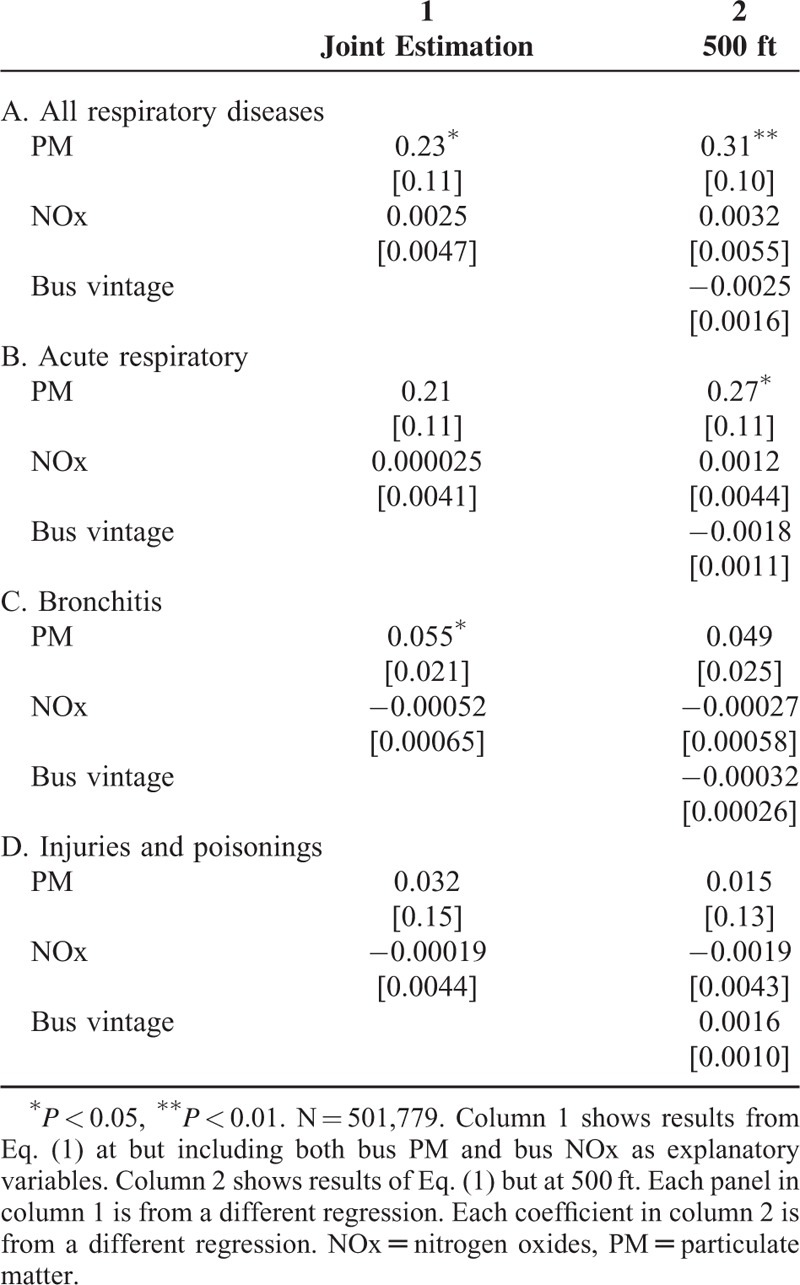
Results From the Sensitivity Analysis Showing Coefficients From the Joint Estimation of Bus PM and Bus NOx and Results From the Static Model at 500 ft

Next, in using Eq. (1) a linear relationship is assumed between health and bus pollution, when it may be nonlinear. To address this, linear splines are used to allow coefficients to vary between breakpoints. Two break points are used within the data using the command “mkspline” in STATA 13.^20^ Results are in Table S3, http://links.lww.com/MD/A418 of the supplementary file and show a significant positive relationship with ED visits for all and acute respiratory diseases from exposure to low levels of bus PM.

## DISCUSSION

Findings in this study are the first to show that stricter EPA bus emission standards are associated with small, but positive health outcomes. Baseline results from the static model in Eq. (1) show that increases in bus PM have a positive contemporaneous effect on ED visits for respiratory disease. There are also important differences by demographic groups, where impacts of bus pollution were exacerbated among Blacks, though no statistically significant effect was found among Whites. There is not enough information to ascertain the reasons for this, but it could be due to socioeconomic factors or practicing avoidance behavior by staying indoors, which has been found in the previous work.^22^ The latter may also explain why there is no effect on more vulnerable populations, like the elderly or very young.

The relationship between bus vintage and background pollution levels is also explored to determine the importance of bus vintage and emission standards on bus pollution. Results show that newer, cleaner buses are associated with statistically significant decreases in background levels of NOx and EC. Though there is no effect on background levels of PM_2.5_, EC is a constituent of PM_2.5_ that is often used as a measure of diesel exhaust particles, in which case EC should better capture diesel emissions from buses. Additionally, the impacts on background EC and NOx from bus vintage decreases with bus routes that are further away, as expected. These results reinforce the critical role of bus vintage and suggest that bus vintage is a good approximation of bus emissions.

Results from the sensitivity analysis examining the cumulative 8-day impact of bus emissions on health suggest permanent changes in bus pollution have positive effects on health outcomes. The main findings of this paper are also robust to other tests, including joint estimation of the impacts of bus NOx and PM and exploring nonlinearities. With respect to the latter, although it is surprising that there is no significant relationship from exposure to higher levels of bus PM, it could be due to the small number of high-emitting vehicles in the bus data set.

### Economic Impacts of Bus Pollution Policies

Respiratory disease accounted for 11% of aggregated U.S. hospital costs and was the second most common reason for ED visits for children under 18 in 2010.^23^ A back-of-the-envelope calculation is used to estimate the economic impact of bus emission standards for those who reside within 300 ft of a bus route. The average hospital charge for an ED visit for any respiratory illness in the hospitalization data set was $33,427 (standard deviation is $63,336). The 8-day cumulative impact from a 0.1 unit increase in bus PM emission standards on daily ED visits for all respiratory illnesses (0.043), as it reflects permanent changes in bus emissions, is multiplied by average charges per ED visit and then by 365 days per year for 4 years. This calculation suggests hospital savings of $2.1 million between 2006 and 2009 from exposure to relatively newer buses.

### Limitations

There are a couple limitations to this study. First, estimated bus PM and NOx likely suffer from the measurement error since the average bus fleet must adhere to emission standards, but not individual buses. However, since the error is random, it would bias estimates toward the null hypothesis and underestimate the true effect. Second, information on smoking or other socioeconomic characteristics of patients are not accounted for. However, as noted earlier, the quasi-experimental regression design minimizes omitted variable bias and results are robust to including census block dummy variables, other important neighborhood characteristics and trends, and results pass the falsification tests, implying it is not a major concern.

Also, the association between exposure to bus PM and health is small which is unsurprising as the period of interest occurs a couple decades after regulations began. Consequently, the estimated economic benefit calculated in this study reflects a lower bound of hospital savings as average bus PM in this study period is 0.05 units, or 92% lower than initial EPA transit bus emission standards. Further, only impacts for those who reside within 300 ft of a bus route, which already includes almost half of NYC residents, are studied. However, given the high population density of NYC, the impacts from bus pollution could extend to residents who live further away. Those who ride the bus, bus drivers, or individuals who work near bus routes are also not considered. Finally, this analysis only accounts for individuals who were sick enough to go the ED for treatment, so individuals who decided to remain at home or who saw their primary care physician instead were not included. As a result, the health and economic benefits resulting from these emission standards are likely many times higher and are evidence that over the past 20 years, bus pollution policies have contributed to critical improvements in public health in urban areas.

## CONCLUSION

This is the first study to rigorously evaluate the relationship between transit bus pollution policies and morbidity, specifically respiratory illness. To circumvent problems of confounding variables, a quasi-experiment using rich panel data is exploited. Findings in this study show robust and consistent improvements in health outcomes resulting from more stringent EPA emission standards for PM. Reconciling these results with estimates showing a negative relationship between bus vintage and background EC levels imply that diesel PM is associated with poorer health outcomes. This study has important implications regarding policy approaches for transportation and sustainability in urban areas. The fact that NYCT embraced these federal policies by phasing out older buses demonstrates the potential for local transit agencies to take an active role in improving urban environmental quality.

## References

[1] American Public Transportation Association (2012). Ridership report: Second quarter Technical Report.

[2] Sydbom A Blomberg A Parnia S Health effects of diesel exhaust emissions. *Eur Resp J* 2001; 17:4733–4746. 10.1183/09031936.01.17407330 11401072

[3] Kagwa J Health effects of diesel exhaust emissions-a mixture of air pollutants of worldwide concern. *Toxicology* 2002; 181-182:349–353. 12505335 10.1016/s0300-483x(02)00461-4

[4] Metropolitan Transit Authority New York City Transit. New York City Transit and the Environment. http://web.mta.info/nyct/facts/ffenvironment.htm/#clean/_bus Accessed January 2, 2012.

[5] NYC Department of Health and Mental Hygiene. New York City Community Air Survey: Results from winter monitoring 2008–2009, 2009.

[6] Beatty T Shimshack J School buses, diesel emissions and children's health. *J Health Econ* 2011; 30:987–999. 21741102 10.1016/j.jhealeco.2011.05.017

[7] Parent ME Rosseau MC Boffetta P Exposure to diesel and gasoline engine emissions and the risk of lung cancer. *Am J Epidemiol* 2007; 165:53–62. 17062632 10.1093/aje/kwj343

[8] Northridge ME Yankura J Kinney PL Diesel exhaust exposure among adolescents in Harlem: a community driven study. *Am J Public Health* 1999; 89:998–1002. 10394306 10.2105/ajph.89.7.998 PMC1508854

[9] NYC Department of Health and Mental Hygiene (2011) Air pollution and the health of New Yorkers: The impact of fine particles and ozone.

[10] X. (2014) Transit buses and fetal health: The impacts of bus pollution policies in New York City. Working paper.

[11] Pope CA III Respiratory disease associated with community air pollution and a steel mill, Utah Valley. *Am J Public Health* 1989; 79(5). 10.2105/ajph.79.5.623 PMC1349506 2495741

[12] Dominici F Greenstone M Sunstein CR Particulate matter matters. *Science* 2014; 344.

[13] Chen Y Ebenstein A Greenstone M Li H Evidence on the impact of sustained exposure to air pollution on life expectancy from China's Huai River Policy. *Proceedings of the National Academy of Sciences*, 2013:110 (32). 10.1073/pnas.1300018110 PMC3740827 23836630

[14] Canagaratna M Jayne J Ghertner D Chase studies of particulate emissions form in-use New York City vehicles. *Aerosol Sci Technol* 2004; 38:555–573.

[15] Clark N Kern J Atkinson C Factors affecting heavy-duty diesel vehicle emissions. *J Air Waste Manag* 2002; 52:84–94. 10.1080/10473289.2002.10470755 15152668

[16] M.J. Bradley and Associates. MTA New York City Transit Bus Fleet Emissions, 1995–2006, 2006.

[17] New York City (NYC) Dept. of City Planning. BYTES of the Big Apple. Accessed March 1, 2012: http://www.nyc.gov/html/dcp/html/bytes/applbyte.shtml.

[18] ESRI 2014. ArcGIS Desktop: Release 10. Redlands, CA: Environmental Systems Research Institute.

[19] Zhu Y Hinds W Kim S Study of ultrafine particles near a major highway with heavy-duty diesel traffic. *Atmospheric Environ* 2002; 36:4323–4335.

[20] StataCorp LP. Stata Statistical Software, Release 13. College Station, TX: StataCorp LP;2014.

[21] Wooldridge J Econometric Analysis of Cross Section and Panel Data. Cambridge, MA: MIT Press; 2002.

[22] Neidell M Air quality warnings and outdoor activities: evidence from Southern California using a regression discontinuity design. *J Epidemiol Community Health* 2010; 64:921–926. 19822555 10.1136/jech.2008.081489

[23] Wier L. M. Yu H. Owens P. L. Washington R. (2013) Statistical brief #157: Review of children in the emergency department, 2010 Agency for Healthcare Research and Quality's Healthcare cost and utilization project. 24006551

